# Comparison of patient flow and provider efficiency of two delivery strategies for HPV-based cervical cancer screening in Western Kenya: a time and motion study

**DOI:** 10.1080/16549716.2018.1451455

**Published:** 2018-03-28

**Authors:** Easter Olwanda, Jennifer Shen, James G. Kahn, Katelyn Bryant-Comstock, Megan J. Huchko

**Affiliations:** a Center for Microbiology Research, Kenya Medical Research Institute, Nairobi, Kenya; b Institute for Health Policy Studies, University of California, San Francisco, CA, USA; c Duke Global Health Institute, Duke University, Durham, NC, USA; d Department of Obstetrics and Gynecology, Duke University, Durham, NC, USA

**Keywords:** Cervical cancer, HPV testing, time and motion, Kenya, CHCs, Clinics

## Abstract

**Background**: Improving patient flow and reducing over-crowding can improve quality, promptness of care, and patient satisfaction. Given low utilization of preventive care in low-resource countries, improved patient flows are especially important in these settings.

**Objective**: Compare patient flow and provider efficiency between two cervical cancer screening strategies via self-collected human papillomavirus (HPV).

**Methods**: We collected time and motion data for patients screened for cervical cancer in 12 communities in rural Migori County, Kenya as part of a larger cluster randomized trial. Six communities were randomized to screening in community health campaigns (CHCs) and six to screening at government clinics. We quantified patient flow: duration spent on each active stage of screening and wait times, and the number of patients arriving at CHCs and clinics each hour of the day. In addition, for four CHCs, we collected time and motion data for providers, and measured provider efficiency as a ratio of active (service delivery) time to total time spent at the clinic.

**Results**: Total duration of screening visits, at CHCs and clinics was 42 and 87 minutes, respectively (p < 0.001 for difference). Total active time lasted longer at CHCs, with a mean of 28 minutes per patient versus 15 minutes at clinics, largely due to differences in duration for group education (p < 0.001). Wait time for registration at clinics was 36 minutes, explaining most of the difference between settings, but sometimes incorporated other health services.

**Conclusions**: There is a substantial difference in patient flow at clinics compared to CHCs. Shorter duration at CHCs suggests that the model is favorable for patients in limiting time spent on screening. Future cervical cancer screening programs designed for scale-up should consider how this advantage may enhance satisfaction and uptake. For clinic-based screening programs, efforts could be made towards reducing registration wait times.

## Background

Health facilities throughout the world face challenges providing care to large volumes of patients efficiently. Improving patient flows and reducing over-crowding are important issues, as research shows inefficient patient flows in clinics affect quality and timeliness of care, safety, and patient satisfaction [1–3]. The lower access to funding, human capital, and infrastructure in low-resource countries make optimizing patient flows essential, but also potentially more challenging in these settings. Ideally, clinics in low-resource settings would be able to maximize the efficiency and minimize costs of patient care. However, health costs in low-resource settings are still poorly understood, which impedes optimal resource allocation [4]. Further research that identifies and measures patient flows and efficiency in low-resource settings will help health systems seeking to increase access to care without increasing the availability of resources.

Only a few studies in Sub-Saharan Africa have measured patient flow in clinical settings. In Ghana, Best et al (2014) used computer simulation to improve patient flow in an acute care hospital. They found that the largest reduction in patient time spent in the hospital occurred when shift times were coordinated with patient arrival patterns [5]. Wanyenze et al (2010) measured patient flows at three antiretroviral therapy (ART) clinics in Uganda in order to identify the clinic with the shortest wait time for patients at the clinic [6]. The researchers also concluded that streamlining doctor and counselor activity could have an effect on patient flow. Another study in Uganda found that task shifting from doctors to nurses and pharmacy-only visits led to reductions in total wait time [7]. Scheduled appointment times with longer appointment times have been shown to reduce wait times [8].

Patient flow analysis is a quality improvement tool to help healthcare facilities identify inefficiencies in patient flow, and provide suggestions for how to intervene and improve processes. In recent years, there have been efforts to systematize and improve methods of patient flow analysis in resource-limited settings. Dixon et al (2015) describe the step-wise approach to patient flow analysis in developing country settings, and apply their method of patient flow analysis to a hospital in Ghana. They found that the average duration of hospital visits was 2 hours longer than the average duration in emergency departments in the USA. The stepwise approach termed the 3Ps – preparation, piloting, and performing – is used as a flexible guide of items to consider when performing a patient flow analysis. After performing a patient flow analysis, the collected data is analyzed as it relates to the objectives and process measures to help identify areas for improvement [9].

Cervical cancer screening is one area where patient efficiency may play a key role in the successful implementation of health care programs for large numbers of women at low-costs and with limited staffing. Cervical cancer is the fourth most common cancer among women in the world, with over 250,000 deaths and 500,000 new cases in 2012 [10]. Furthermore, more than 80% of cervical cancer cases occur in the least developed countries [11]. Human papillomavirus (HPV) causes about 70 percent of all cases of cervical cancer, and studies in low-resource settings support the cost-effectiveness of HPV-based cervical cancer screening [12]. Based on a systematic review of randomized control trials and observation studies, the World Health Organization (WHO) issued a new set of guidelines in 2013 for cervical cancer screen-and-treat techniques, and recommends the HPV test followed by treatment with cryotherapy for women who test positive [13].

A large majority of worldwide cervical cancer burden is in less-developed regions, at about 85%. The annual number of cervical cancer cases in Sub-Saharan Africa in 2012 was about 92,000 [14]. Despite new strategies to increase availability of screening and treatment of cervical pre-cancer, screening rates remain low in Sub-Saharan African countries [15]. From a provider survey implemented in rural Kenya, Rosser et al (2015) found barriers to screening from the patient perspective including wait time, discomfort with male providers, and inadequate knowledge and counseling skills. Providers also cited shortages of staff and lack of space as barriers to screening. Long wait times at clinics are common in Sub-Saharan African countries, where clinics have limited infrastructure and human resources [16]. A study at Mulago Hospital in Uganda found that a very high outpatient load was overwhelming staff and resources, resulting in long wait times and poor patient satisfaction. [17] A main driver of the lower efficiency of healthcare service delivery is overextended staff, leading to longer wait times for the consultation, and slower dispensing of drugs and laboratory tests [18].

Cervical cancer screening programs in low-resource settings need to reach large numbers of women, and must include educational programs and counseling to increase acceptability and reduce stigma. Despite simplified screening strategies, educational programs may increase duration of time spent at clinics when receiving services [19]. Research is needed on clinical care processes that minimize total time spent at the clinic for cervical cancer screening. As low and middle-income countries establish and expand cervical cancer programs, few studies have examined the flow of patients, the sources of bottlenecks and potential avenues for improving patient flow and quality of care. An understanding of patient flow will be essential for sustaining HPV self-sampling scale-up and progressing toward universal access to cervical cancer screening. In this study, we conducted a patient flow analysis of two models of delivering cervical cancer screening via HPV self-collection using time and motion data. The purpose was to inform improvement of patient flow for cervical cancer screening services in low-resource countries.

## Methods

We used time and motion data to estimate the duration of screening and identify inefficiencies in two models of cervical cancer screening with self-collected HPV in a rural county in western Kenya: (1) screening offered through short-term community health campaigns (CHCs), where women were screened at temporary tents set up close to community centers, and (2) screening integrated into government health clinics. The goals were to identify bottlenecks in cervical cancer screening patient flow, and mismatches between screening demand and provider staffing.

### Study description

A time and motion study is a method used to characterize the efficiency of service delivery [20]. The time and motion study involved systematic observation of all daily tasks carried out in the clinic and CHCs. We measured the time spent on each of the daily tasks observed at clinics and CHCs, and used the data collected to measure standard times required to perform all aspects of screening. Previous studies have validated time-and-motion methodologies within clinical settings to improve procedures, and increase productivity [21,22].

This time and motion study was conducted as part of a cluster-randomized clinical trial in 12 communities in Migori County, in Nyanza, Kenya between January and September 2016. The target population was women eligible for cervical cancer screening per the Kenya Ministry of Health guidelines: 25–65 years old with an intact uterus and cervix. Six communities were randomized to offer screening in CHCs and the remaining six communities provided screening at government clinics. Huchko et al (2017) reported the main outcomes of the trial, which showed that cervical cancer screening offered in CHCs reached a larger proportion of women in study communities [23].

In the CHC model, mobile screening activities were held at up to 10 sites within each community over a period of two weeks. Sites selected for the mobile CHC included church compounds, schools, open fields, and market centers. The CHC tent had a partitioned area for self-collection, a registration table, and a group education area. During the day, community health volunteers (CHVs) conducted door-to-door mobilization under the supervision of two program assistants. Samples were processed and analyzed using the careHPV system which was located at a laboratory at Migori County Hospital. The care HPV system provided a positive result if at least one of the 14 high-risk genotypes of HPV was identified.

**Figure 1.**
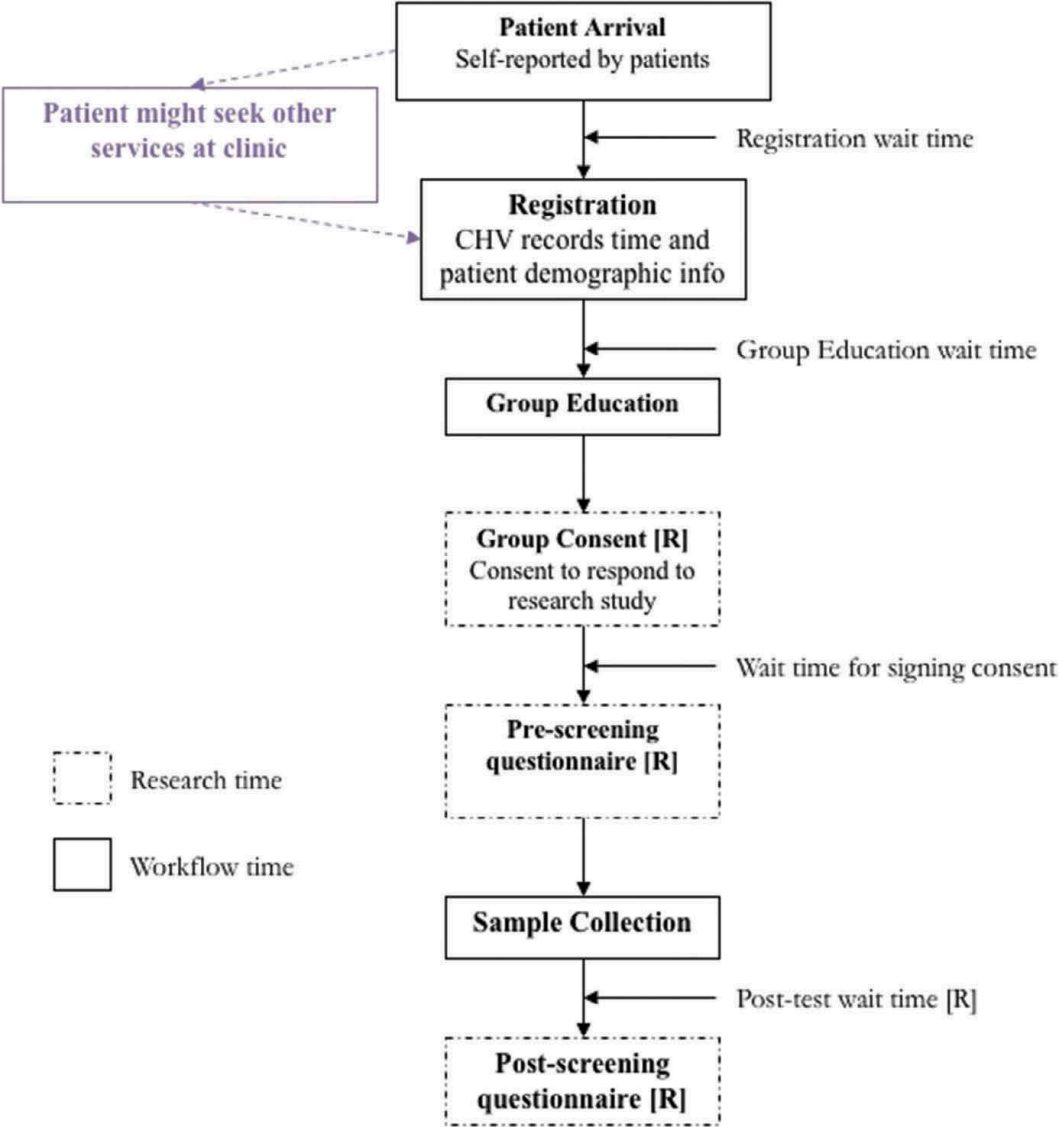
Diagram of patient flow steps at CHCs and clinics for cervical cancer screening.

At clinics, the screening workflow was similar to the CHC except that all steps were performed by a single CHV. The women could have been visiting the clinic for other health services or just for cervical cancer screening. The CHV registered the patients, facilitated individual or group education and consent, administered the pre-test survey, directed patients to self-collection rooms, and administered the post-test survey. See Figure 1 for the workflow for CHCs and clinics.

At all 12 communities, women screened for HPV through self-test kits. Self-testing for HPV, which has similar sensitivity and specificity to samples taken by clinicians [24,25], has the potential to address many barriers to screening and increase the participation rates of at-risk and hard-to-reach women [26].

### Data collection

We collected patient time and motion data to measure time spent at each stage of cervical cancer screening at each of the 12 CHCs and clinics. Provider time and motion data were collected at four CHCs.

Patient time and motion measures collected were total visit time, time at each stage of the screening process, wait time between each stage of screening, and patient arrival patterns. Provider time and motion included provider productive (service delivery) and idle time. The method of data collection differed slightly between CHCs and clinics. At CHCs, research assistants recorded the amount of time spent on all activities involved with screening, from the patient’s arrival to the end of the post-test questionnaire, using activity forms with time stamps, programmed on OpenDataKit (ODK) software.

At the beginning of a patient’s visit to the CHC, research assistants recorded the patient’s arrival time, and registration start and stop time. The patient then moved to participate in group education. The start time for group education depended on the next available sizeable group of participants. After group education, the patients would move to a private area where they would individually sign consent forms with research assistants. Patients were then issued HPV self-collection kits and administered a pre-test questionnaire. The patients entered sample collection rooms and proceeded with specimen collection. After sample collection was completed, patients would fill out a post-test questionnaire again administered by the research assistant.

At the clinics, CHVs recorded time spent on paper forms, which were later entered into the ODK software. The main difference for screening at clinics was the possibility that patients received other services while they waited for screening services. Education session wait time was also dependent on the availability of the CHVs, who were often involved with other clinic activities. Education sessions could be done individually or in groups, depending on the patient volume. At clinics, data quality checks were put in place to ensure that data recorded by the research assistants at each of stage of the screening procedure were consistent across patients.

After both clinic and CHC data were entered into ODK, we uploaded the data to an ODK Aggregate server, and imported the data into SAS for management (merging, cleaning, sorting) and reporting. We exported data from SAS to MS Access for further cleaning and storage in MS Excel format. The costing team which comprised the costing lead and the costing assistant transferred the data into clinic and CHC time and motion Excel workbooks for analysis.

Figure T.S. 1 in the Technical Supplement shows the time and motion log used by the research assistants and CHVs, either electronically or paper-based.

### Analyses

To understand the bottlenecks in cervical cancer screening in the two clinical models, we descriptively analyzed the time-and-motion data to measure differences in patient arrival numbers and patient and provider time measures. We estimated the number and timing of patients arriving at CHCs and clinics to understand the distribution of activity in an average day. Patient arrival numbers were measured as the number of patients arriving at the CHC or clinic during each hour of operation. Patient time measures included total visit time and total wait time. Visit time was composed of time at each stage of the screening process and wait times between each stage.

We also measured and compared averages of provider times for CHCs, using the provider time-and-motion data. These included active time (i.e. amount of time spent on productive clinic/CHC activities) and idle time (i.e. amount of time spent at the CHC/clinic waiting).

The final analysis was a comparison of patient and provider efficiency. Patient efficiency was measured as the amount of active time (i.e. time spent at the CHC excluding wait times) divided by the total time spent at the CHC. Provider efficiency was measured as the amount of active time (i.e. time spent working at the CHC excluding idle time) divided by total working time at the CHC. We hypothesized that there was a tradeoff between patient efficiency and provider efficiency, meaning that if there were more providers at the CHC, then there was a lower likelihood that patients needed to wait for each activity resulting in a high patient efficiency. With more providers at a CHC, we hypothesized there was a higher likelihood that providers were idle, which meant lower provider efficiency.

## Results

Between January and September 2016, 2899 women were screened at the six CHCs, and 2042 women were screened at clinics. Table 1 compares average time durations for screening at CHCs and clinics, broken down by total active and wait times. The total duration of screening, excluding research time, at CHCs was 42 minutes and at clinics was 1 hour and 27 minutes. The difference was statistically significant (p < 0.001, t-test = 25.44). Visiting a CHC for screening saved a patient about 45 minutes. Total activity time (i.e. time spent on stages related to screening, excluding wait time) lasted longer at CHCs, at 28 minutes on average per patient compared to 15 minutes at clinics (p- = 0.00, t-test = 21.00).

**Table 1.** Average duration for total visit, wait time, and stages of screening, per patient, for CHCs and clinics.

HH:MM per patient	CHCs	Clinics	p-value
Total duration of screening	0:42	1:27	<0.001
	(0:23)	(1:19)	
Total activity time	0:28	0:15	<0.001
	(0:18)	(0:10)	
Total wait time	0:15	1:12	<0.001
	(0:13)	(1:17)	
Total wait time excl. registration	0:14	0:36	<0.001
	(0:11)	(0:56)	
Registration wait time	0:01	0:36	<0.001
	(0:05)	(0:53)	
Registration	0:03	0:02	0.20
	(0:15)	(0:06)	
Group education wait time	0:07	0:22	<0.001
	(0:06)	(0:49)	
Group education	0:19	0:09	<0.001
	(0:08)	(0:07)	
Sample collection	0:05	0:04	<0.001
	(0:03)	0:02	
Time spent on research	0:26	0:17	<0.001
(includes wait time)	(0:12)	(0:11)	

Total duration of screening excludes any activity or wait time related to research. Standard errors in parentheses.

Wait times were extremely long for clinics (36 minutes), reflective of the fact that some patients at clinics may have received other health services before registering for cervical cancer screening. When excluding registration, the difference in duration spent at clinics and CHCs was reduced to 22 minutes (p < 0.001), suggesting that the difference in wait times could be mostly attributed to the longer wait times for registration.

While group education wait time was considerably longer for clinic (22 minutes) compared to CHCs (7 minutes), active time in group education lasted longer at CHCs. Counseling on cervical cancer screening at CHCs may have therefore been more comprehensive, or the group setting may have facilitated more questions. Sample collection lasted slightly, but significantly, longer at CHCs, at 5 minutes compared to 4 minutes at clinics (p-value< 0.001). All research activities (group consent, pre-screening questionnaire, post-test wait time, and post-screening questionnaire), were aggregated together for both CHCs and clinics. Research time lasted longer at CHCs, at 26 minutes compared to 17 minutes at clinics, and this was statistically significance (p-value<0.001).

**Figure 2.**
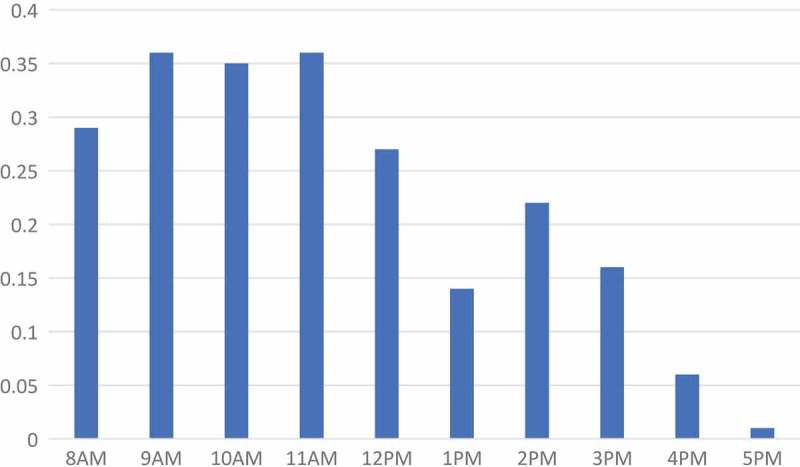
Number of patients arriving to clinics per hour, averaged across community-days.

Figure 2 shows the number of patients that arrived to clinics per hour, averaged across community-days. On average the number of patients that arrived each hour at clinics was less than one patient. The range in average number of patients that arrived during an operating hour was 0.01 to 0.36. The highest amount of activity at clinics was in the morning, between 9am and 12pm; the patient arrival rate declined gradually after 12pm.

**Figure 3.**
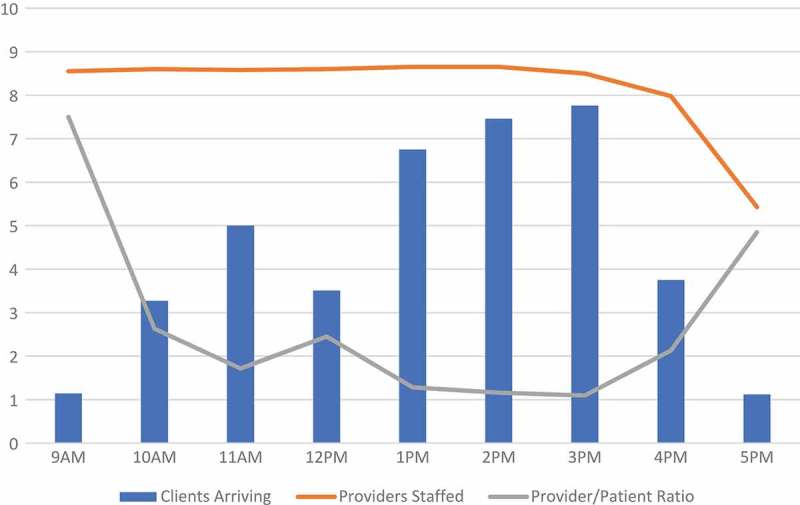
Number of patients arriving, providers staffed, and provider-patient ratio by hour, at CHCs, averaged across community-days.

The distribution of patients arriving at CHCs was different in size and shape relative to clinics. In Figure 3, we can see that the busiest hours for CHCs were from 1 to 4pm. CHCs were also clearly busier than clinics, where the average number of patients that arrived in a given hour ranged from less than one to slightly less than eight patients between 3 to 4pm. We overlaid the number of providers staffed at CHCs each hour, on average. The number of providers engaged in patient care remained high from 8am to 4pm, with a few engaged at the CHCs between 4 and 6pm. While numbers of providers staffed suggests that there may have been an oversupply of providers in the mornings at CHCs, the ratio of providers to patients is fairly stable, remaining between 1 and 3 per patient arriving in an hour (besides at 9am and 5pm which are the starting and close times).

### Comparison of patient efficiency and provider efficiency

Table 2 shows the average provider time spent active, idle, and on research in a day at a CHC. Provider active time is on average 2 hours, idle time is 4 hours and 16 minutes, and research time is an hour and 26 minutes. The total provider time spent at a CHC on average is slightly less than 8 hours.

**Table 2.** Provider time spent on activity, idle, and research, averaged per provider per day.

HH:MM per provider per day	CHCs
Active Time	2:03
	(2:10)
Idle Time	4:16
	(2:07)
Research Time	1:26
	(1:44)
Total Duration	7:45
	(1:43)

CHCs lasted for 10 working days. Total number of observations at the provider-day level was 347. Total number of providers across the four CHCs was 18.

**Figure 4.**
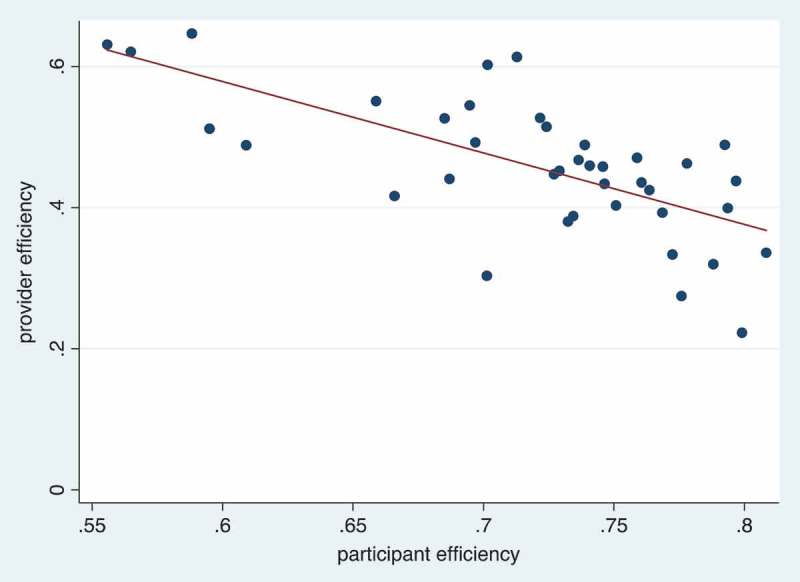
Scatter plot of provider efficiency (i.e. ratio of active time to total work time) and patient efficiency (i.e. ratio of active time to total time spent at CHC) for each community-day, across 4 CHCs, with line of best fit.

In Figure 4, we compare provider efficiency and patient efficiency at CHCs in a scatterplot. We use the provider measures in Table 2 to estimate provider efficiency. Each observation in the scatterplot represents the estimated provider efficiency in a day relative to the estimated patient efficiency in a day. There are a total of 40 observations in the scatterplot from the four CHCs with time-and-motion data. The line of best fit through the scatterplot shows that there was a clear negative relationship between provider efficiency and patient efficiency, which confirms our hypothesis that with improved patient efficiency, there is a higher likelihood that providers end up being idle.

## Discussion

The results of this study provide some insights into the processes and efficiencies of implementing cervical cancer screening in low-resource, rural areas. The first finding is that cervical cancer screening at CHCs provided a quicker and more efficient experience for women than screening at clinics, and the time savings was largely due to differences in wait times. A study conducted in Uganda also demonstrated that CHCs provided a rapid approach to testing a majority of residents for HIV in rural African settings [27]. The CHCs in our study achieved the target adopted by the Kenya Ministry of Health patient charter with overall wait times at 15 minutes, compared to the 20 minutes charter target [28]. Time savings have been identified as an important opportunity cost that should be measured, valued, and included in cost-effectiveness analyses of cervical cancer screening [29].

Our results also show that clinics, had longer registration wait times than CHCs. A study on patients in Malaysia also showed that patients experienced long wait times to register to see a doctor [30]. The main reason for late registration was due to inadequate staff. In our study, long wait times at clinics was driven by patients’ need to re-register and wait for other services like maternal and child health services. Clinics could improve efficiency by reducing registration wait time through integration of cervical cancer screening as part of a larger package of maternal services; for example, integrating with HIV testing and care. One study in Zambia found that increasing linkages and improving integration of HIV and sexual and reproductive health services led to more cost-effective service delivery [31].

The total difference in wait time between CHCs and clinics, excluding the registration wait time, was still large (22 minutes) and significant. Community health volunteers may have worked at maximum capacity to service a larger than expected number of patients, possibly resulting in the long wait times post-registration. Another possible solution is for clinics to ensure that a greater number of trained staff are available during peak hours, and schedule for staff downtime during periods of low demand. A similar recommendation has been previously made to counter this challenge in outpatient settings [32].

Another finding from the study was that total activity times (time spent on screening-related tasks) were shorter at clinics, and this difference was most pronounced in the differences in time spent on group education: CHCs spent on average 19 minutes on group education, and clinics spent on average 9 minutes. From the study protocol, the group education module takes approximately 15 minutes to administer [33]. At clinics, since CHVs were required facilitate the entire screening process, the CHV likely focused on patient volumes and the need to attend to all within the available time. The CHV therefore may have shortened time spent on group education. The other possible explanation is that since there were lower volumes of women screening at any given time at the clinics, group education lessons moved more quickly. If CHVs, indeed, had less time to spend with their patients because of issues of capacity, then women had less time to ask the provider questions and better understand the process of screening and testing. Addressing provider capacity at clinics therefore could increase the necessary time spent to provide care to patients, help patients understand the screening process and subsequent testing procedures, and reduce the amount of wait time spent at clinics.

Another finding was the relationship between provider staffing and number of patients arriving for cervical cancer screening services. From our analyses of patient arrivals and provider staffing at CHCs, we found that the number of providers relative to patients arriving at the clinic were slightly higher in the mornings than in the afternoons, although provider-patient ratios did not exceed 3 between 10am and 4pm. We also found that there was a tradeoff in provider efficiency (i.e. ratio of active time to total time working) and patient efficiency at CHCs (i.e. ratio of active time to total time spent at the clinic). For future CHCs that will be implemented for cervical cancer screening in low-income, rural settings, there are benefits for patients to have higher provider-patient ratios because more providers staffed means lower likelihood of long wait times at the CHC. With the goal of achieving more efficient allocation of resources for cervical cancer screening programs, future implementers could also consider more efficiently staffing providers based on projected numbers of patients who arrive at the clinic or CHC for screening.

Finally, both cervical cancer screening models implemented self-testing for HPV, which is an attractive option for other cervical cancer programs because of the convenience and time-efficiency. Women in other contexts have reported that self-testing helped overcome long wait times at the clinic and thus reduced the amount of time spent away from work [34]. More recently, self-testing for HPV has become more common in low and middle-income countries, and governments are in the process of considering expanded programs and different delivery models for HPV-screening. In doing so, considerations of the optimal implementation model should include patient visit time and the implications of staffing levels on provider and patient efficiency.

## Limitations of the study

Our study has several limitations. The first was that women at clinics who were waiting to register for screening often received other services besides cervical cancer screening during their time waiting. We do not have data on whether women attended clinic solely for cervical cancer screening. Because women self-reported their arrival times, the study team may have included the time spent on other services as registration wait time, which extended the recorded wait times for registration. Therefore, wait times recorded may not be an accurate measure of pre-screening wait, although total duration at the clinic is accurate. Future studies should document and incorporate evaluation of whether women received other services while at the clinic when waiting for screening services. Another limitation is that provider time and motion data was collected in four CHCs only. We did not collect time-and-motion data in the first two communities to ensure that we were collecting data from CHCs with resolved implementation issues. However, this prevented a larger sample to measure the relationship between provider efficiency and patient efficiency, and also prevented comparison with clinics. The final limitation is that while we did find shorter time durations spent on cervical cancer screening at CHCs, each CHC required a larger team to implement screening activities, and the availability of screening in communities only lasted for two weeks. Implementers and policymakers considering the expansion of cervical cancer screening in low-resource countries need to consider these tradeoffs when making decisions on screening models.

## Conclusion

This is the first study to compare patient flows for different cervical cancer screening models in a rural area of a low-resource country, to our knowledge, and one of few patient flow studies in these settings. Our results inform health systems and program implementers on how to successfully sustain and scale programs in HPV self-sampling, and influence further progress toward universal access of cervical cancer prevention.

## Recommendations

Future studies could explore the effects of reducing clinic durations by consolidating registration across other health care services or adjusting provider staffing at CHCs based on projected number of patients arriving at the CHC. Importantly, future studies should explore the patient perspective and experience with various visit lengths and wait times.

## Paper context

Improving patient flow in clinics located in low-resource settings is important for expansion and sustainability of healthcare programs. Few studies have measured patient flow in low-resource settings. We compared screening durations and staff efficiency of two models of cervical cancer screening in Western Kenya and find shorter patient screening times for the community-based screening strategy. We recommend more attention towards improving patient flow, and further consideration of efficiencies offered by community-based screening.
