# Supplementary material for: Comparison of patient flow and provider efficiency of two delivery strategies for HPV-based cervical cancer screening in Western Kenya: a time and motion study
Source: Glob Health Action. 2018 Mar 28;11(1):1451455. doi: 10.1080/16549716.2018.1451455 (PMC5912439; doi:10.1080/16549716.2018.1451455)
Supplement: Supplementary material [file ZGHA_A_1451455_SM6330.zip › Figure TS 3.docx]

**Figure T.S. 3:** Registration form for patients arriving at CHCs (similar at clinics)
